# Research on the Mass Adding and Removing Combined Mechanical Trimming Method of the Ring MEMS Gyroscope

**DOI:** 10.3390/mi14101957

**Published:** 2023-10-20

**Authors:** Xinyu Wang, Kai Wu, Chengxiang Wang, Qingsong Li, Zhanqiang Hou, Dingbang Xiao, Xuezhong Wu

**Affiliations:** 1 College of Intelligence Science and Technology, National University of Defense Technology, Changsha 410073, China; xyw22@nudt.edu.cn (X.W.); wukai@nudt.edu.cn (K.W.); cx.wangnudt@nudt.edu.cn (C.W.); houzhanqiang@nudt.edu.cn (Z.H.); dingbangxiao@nudt.edu.cn (D.X.); xzwu@nudt.edu.cn (X.W.); 2 Hunan MEMS Research Center, Changsha 410073, China

**Keywords:** MEMS gyroscope, frequency splitting, mechanical trimming, femtosecond laser

## Abstract

The MEMS gyroscope is one of the basic units of inertial navigation, whose performance and accuracy is noteworthy. Because of the limitations of processing technology and other factors, the relative manufacturing error of MEMS gyroscopes is usually large. Errors directly lead to a frequency mismatch of resonant structures and consequently restrict the performance improvement of the gyroscope. This study proposes a mechanical trimming technique combining the addition and removal of gold in a ring MEMS gyroscope. Firstly, the analysis of the gyroscope dynamics and error model and trimming theory provides theoretical guidance for the trimming process. Secondly, the method of adjusting the mass is investigated, and the ablation threshold of femtosecond laser parameters on gold is analyzed, which provides the process with parameters for the trimming experiment. Finally, the frequency trimming process is conducted in three steps, including the addition of gold spheres and the removal of gold spheres and gold film, which are applicable to the trimming process at different rates of frequency split. The results shows that the proposed method can reduce the frequency split of the gyroscope from 4.36 to 0.017 Hz.

## 1. Introduction

MEMS gyroscopes, which are used to measure angular velocity and angular displacement, are widely applied in the fields of aerospace, automotive, and industrial automation due to their small size, light weight, and low power consumption [1,2,3]. However, because of its limitations in manufacturing processes, materials, and other factors, the relative error rate of the gyroscope is usually large [4]. Errors result in frequency mismatch, which reduces the performance of the gyroscope, including zero bias stability and sensitivity [5,6]. Therefore, it is important to pay attention to frequency matching and trimming technology when using such a gyroscope. Frequency mismatch can be reduced in the design and processing stage. Meanwhile, frequency splitting is usually controlled via two methods—electrostatic and mechanical trimming [7,8]. Electrostatic trimming shows excellent performance in the accuracy of trimming, but it is not steady, since its performance is susceptible to variations in temperature, and its trimming ability is limited [9]. Therefore, it is important to study mechanical trimming because of its stability, reliability, and environmental adaptability.

Mechanical trimming techniques for vibrating gyroscopes have been widely investigated. Regarding mass-adding trimming, Kim et al. employed a precision solder sphere with a diameter of 75 μm or a silver nanoparticle ink droplet with a diameter of 40 μm. Their results show that the frequency mismatch of the disk gyroscope is reduced from 14.2 to 0.07 Hz [10]. Ma et al. decreased frequency splitting from 10 to 1 Hz via attaching mass blocks to a bell gyro [11]. Regarding mass-removing trimming, Viswanath et al. proposed a high-resolution micro-ultrasonic machining (HR-μUSM) process, which is used for the post-processing trimming of complex 3D microstructures that are made of fused silica. The HR-μUSM process achieves the mass removal of the sub-nanometer surface [12]. Hu et al. determined the processing parameters of etched holes via finite element simulation and applied a femtosecond laser to manufacturing holes. The gyroscopic frequency mismatch is reduced from 2.090 to 0.119 Hz [13]. In general, it is difficult to control the accuracy of the added mass and trimming when mass-adding trimming is applied alone. Similarly, mass-removing trimming also shows its disadvantage. The quality factor of the structure is influenced by damage to the original structure, and the trimming ability of the method is also insufficient. Therefore, it is more likely that the performance of the mechanical trimming will be improved by combining the mass adding and removing methods.

This study focuses on the ring MEMS gyroscope, which is made of fused silica. A combined method is proposed in which the mass is first added and then removed based on the mass carrier of gold. The method employs a femtosecond laser to ablate the added gold without damaging the original structure based on proper process parameters. The result shows that the frequency splitting of the gyroscope is reduced from 4.36 to 0.017 Hz.

## 2. Working Principle of the Gyroscope and Analysis of Frequency Splitting

### 2.1. Working Principle

**Figure 1:**
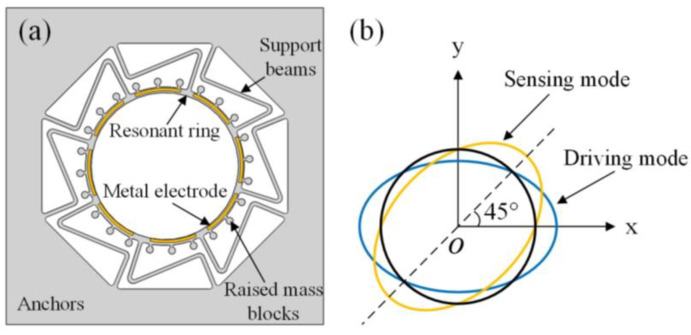
(**a**) Resonant structure; (**b**) the *n* = 2 working mode.

MEMS gyroscopes can detect angular velocity or angular displacement based on the Coriolis effect in the resonant state. The resonant structure of the gyroscope includes support beams, a resonant ring, and 24 raised mass blocks, as shown in Figure 1a. The mass distribution of the structure can be changed without affecting the stiffness at the positions of mass blocks. Consequently, the frequency trimming of mass–stiffness decoupling can be realized [14]. The upper surface of the resonant ring contains metal electrodes, while a magnetic field perpendicular to the electrodes is present, making vibration drive and signal sensing possible on the plane along the radial direction according to the laws of ampere force and electromagnetic induction.

Gyroscopes have two working control modes. In the rate mode, the gyroscope is subjected to angular velocity, and the energy is coupled from the main mode to the sensitive mode. By detecting the vibration displacement amplitude in the direction of the sensing axis, angular velocity can be obtained. In the rate integration mode, the gyroscopic vibration mode will change with the input angle and perform circumferential self-precession. The feeding angle is proportional to the input angle. Then, the input angle can be determined according to the vibration angle. In both control modes, the ring gyroscope works in n=2 elliptical mode. The working mode contains two sub-modes, which are the driving mode and sensing mode, with the same frequency and 45° difference in vibration angle, as shown in Figure 1b.

### 2.2. Equivalent Lumped Parameter Dynamic Model and Error Model

**Figure 2:**
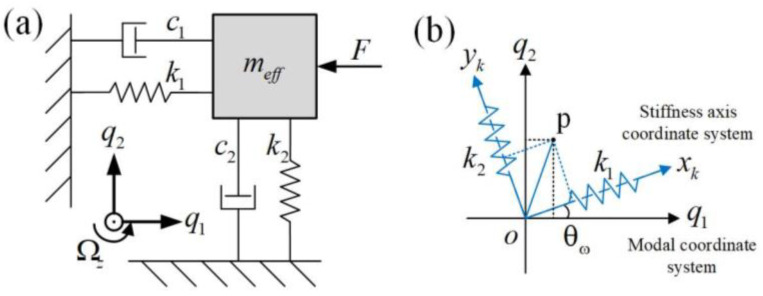
(**a**) Equivalent lumped parameter model; (**b**) the schematic of stiffness asymmetry.

According to the principle of equal energy and consistent natural frequency of the system, the ring gyroscope is illustrated by a two-degree of freedom lumped parametric model (Figure 2a). Here, $q_{1}$ and $q_{2}$ are the displacements of driving and sensing modes, respectively, $m_{eff}$ is the equivalent mass, $k_{i}$ and $c_{i}$ are the equivalent stiffness and equivalent damping, i=1,2; $\Omega_{z}$ is the angular velocity to be detected; and $k_{1}$ and $k_{2}$ are the stiffness of the main stiffness axis. The kinetic equation of the model in the open-loop mode is as follows:

(1) $$\begin{array}{l} m_{eff}{\ddot{q}}_{1}+c_{1}{\dot{q}}_{1}+k_{1}q_{1}=F_{0}\sin(\omega_{d}t)\\ m_{eff}{\ddot{q}}_{2}+c_{2}{\dot{q}}_{2}+k_{2}q_{2}=-4m_{eff}A_{g}\Omega_{z}{\dot{q}}_{1} \end{array}$$

where $A_{g}$ is angle gain, ${\ddot{q}}_{i}$ and ${\dot{q}}_{i}$ are second-order and first-order differentials, respectively, i=1,2, $\omega_{d}$ is the frequency of excitation signal, and t is the time of excitation.

The main stiffness axes of the gyroscope are orthogonal in the mode coordinate system, as shown in Figure 2b. Here, p is a point in the coordinate system. It is worth noting that the position of the main stiffness axis is different from the main vibration mode axis because of the manufacturing process and other factors. In addition, a deviation angle of $\theta_{\omega }$ from the mode vibration axis exists.

In the n=2 working mode of the gyroscope, the clamping angle of the actual main stiffness axis is the same as the vibration axis clamping angle (45°). Along the two stiffness axes, the natural frequency of the resonant structure reaches its maximum and minimum values, respectively. The stiffness axis coordinate system $x_{k}$-o-$y_{k}$ is rotated to the modal coordinate system $q_{1}$-o-$q_{2}$ and the damping asymmetry error is ignored. For ease of description, $q_{1}$ and $q_{2}$ are correspondingly replaced by *x* and *y*. Based on Equation (1), the dynamic equation with errors is shown as follows:

(2) $$\left[\begin{array}{c} \ddot{x}\\ \ddot{y} \end{array}\right]+\left[\begin{array}{cc} \omega^{2}+\omega \Delta \omega \cos(2\theta_{\omega }) & \omega \Delta \omega \sin(2\theta_{\omega })\\ \omega \Delta \omega \sin(2\theta_{\omega }) & \omega^{2}-\omega \Delta \omega \cos(2\theta_{\omega }) \end{array}\right]\left[\begin{array}{c} x\\ y \end{array}\right]+\frac{2}{\tau }\left[\begin{array}{c} \dot{x}\\ \dot{y} \end{array}\right]=\left[\begin{array}{c} F_{0}\sin(\omega_{d}t)\\ -4m_{eff}A_{g}\Omega_{z}{\dot{x}}_{1} \end{array}\right]$$

where τ is the decay time constant, and $\omega^{2}=\frac{\omega_{1}^{2}+\omega_{2}^{2}}{2}$, $\omega \Delta \omega =\frac{\omega_{1}^{2}-\omega_{2}^{2}}{2}$.

### 2.3. Identification of Frequency Splitting and Position of the Main Stiffness Axis

The trimming aims at reducing the frequency splitting on the main stiffness axis. Generally, the natural frequency is affected by both stiffness and mass. However, as mentioned above, the structure investigated in this study is mass–stiffness decoupled. Therefore, it is feasible to only employ the mass trimming method to complete the frequency matching at the position of the main stiffness axis.

In this case, it is necessary to determine the initial frequency splitting and the position of the main stiffness axis.

**Figure 3:**
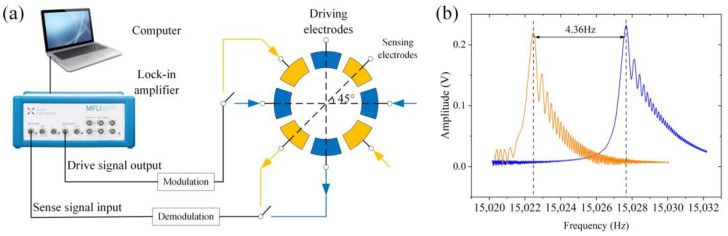
(**a**) Measurement system; (**b**) the identification of frequency splitting.

The details of the measurement method for frequency splitting are described as follows. Firstly, the excitation signals are applied to the driving axis and the sensing axis of the gyroscope by the lock-in amplifier, respectively. Secondly, the signals are detected to obtain the amplitude–frequency curves near the resonance frequency. The frequency splitting is the difference in relative frequency compared to resonant peak positions of the two axes, as shown in Figure 3.

The identification of the position of the main stiffness axis is also completed by analyzing the amplitude–frequency curves. The deflection angle of the main stiffness axis is calculated by the ratio between the resonance peaks. The damping parameters are ignored to simplify the calculation process. According to Equation (2), the free vibration equation of the gyroscope in both axis directions is shown as follows:

(3) $$\left[\begin{array}{c} \ddot{x}\\ \ddot{y} \end{array}\right]+\left[\begin{array}{cc} \omega^{2}+\omega \Delta \omega \cos(2\theta_{\omega }) & \omega \Delta \omega \sin(2\theta_{\omega })\\ \omega \Delta \omega \sin(2\theta_{\omega }) & \omega^{2}-\omega \Delta \omega \cos(2\theta_{\omega }) \end{array}\right]\left[\begin{array}{c} x\\ y \end{array}\right]=0$$

The general solutions of Equation (3) are in the following form:

(4) $$\begin{array}{c} x(t)=A\cos(\omega_{1}t)\cos(2\theta_{\omega })-B\cos(\omega_{2}t)\sin(2\theta_{\omega })=X_{1}-X_{2}\\ y(t)=A\cos(\omega_{1}t)\sin(2\theta_{\omega })+B\cos(\omega_{2}t)\cos(2\theta_{\omega })=Y_{1}+Y_{2} \end{array}$$

Consequently, the declination angle of the stiffness axis can be obtained from the general solutions:

(5) $$\theta_{\omega }=\frac{1}{2}\mathrm{arccot}(\frac{X_{1}}{Y_{1}})=\frac{1}{2}\arctan(\frac{X_{2}}{Y_{2}})$$

(6) $$\theta_{\omega }=\frac{1}{2}\mathrm{arccot}(K\frac{X_{1}}{X_{2}})=\frac{1}{2}\arctan(K\frac{Y_{1}}{Y_{2}})$$

where $X_{1}$ and $Y_{1}$ represent the peak values of the amplitude–frequency curve of the driving axis at the resonance frequencies $\omega_{1}$ and $\omega_{2}$, respectively. $X_{2}$ and $Y_{2}$ are the peak values of the amplitude–frequency curve of the sensing axis at the resonance frequencies $\omega_{1}$ and $\omega_{2}$, respectively. In Equation (6), K=A/B, which is calibrated in the experiment. After calculating the deflection angle of the main stiffness axis, appropriate trimming measures can be determined: that is, adding mass to the high-frequency axis or removing mass from the low-frequency axis.

### 2.4. Trimming Model

After obtaining the aforementioned initial frequency splitting and identifying the positions that require trimming, the amount of mass should be determined. According to the trimming model of C. H. J. Fox [15], when a gyroscope is working in n=2 mode, the position of the high-frequency axis after adding random mass perturbations to the ideal circular ring is shown as follows:

(7) $$\tan(4\theta_{\omega })=\frac{-\lambda_{m}\Sigma_{i=1}^{N_{m}}(m_{i}\sin4\phi_{i})}{-\lambda_{m}\Sigma_{i=1}^{N_{m}}(m_{i}\cos4\phi_{i})}$$

Correspondingly, the frequency also varies.

(8) $$\begin{array}{l} \omega_{1}^{2}=\omega_{0}^{2}\frac{(1+\alpha_{2}^{2})M_{0}}{\left(1+\alpha_{2}^{2})(M_{0}+\Sigma_{i=1}^{N_{m}}m_{i})-(1-\alpha_{2}^{2})\Sigma_{i=1}^{N_{m}}(m_{i}\cos(4(\phi_{i}-\theta_{\omega }))\right)}\\ \omega_{2}^{2}=\omega_{0}^{2}\frac{(1+\alpha_{2}^{2})M_{0}}{\left(1+\alpha_{2}^{2})(M_{0}+\Sigma_{i=1}^{N_{m}}m_{i})+(1-\alpha_{2}^{2})\Sigma_{i=1}^{N_{m}}(m_{i}\cos(4(\phi_{i}-\theta_{\omega }))\right)} \end{array}$$

where $\lambda_{m}=\frac{(1-\alpha_{2}^{2})\omega_{0}}{(1+\alpha_{2}^{2})M_{0}}$, which illustrates the sensitivity of frequency splitting to mass, $\alpha_{2}$ is the ratio of radial amplitude to tangential amplitude, and $m_{i}$ and $\phi_{i}$ are the added mass and position, respectively. According to the derivation completed by Dingbang Xiao et al. [16], the perturbation model can be described using complex vectors.

(9) $$\Delta \exp(j4\theta_{\omega })=-\lambda_{m}\Sigma_{i=1}^{N_{m}}m_{i}\exp(j4\phi_{i})$$

where $\Delta =\omega_{1}-\omega_{2}$, with assuming that $\omega_{1}>\omega_{2}$. Furthermore, since each modification only changes the mass at a certain position, Equation (9) is simplified.

(10) $$\Delta \exp(j4\theta_{\omega })=-\lambda_{m}m_{1}\exp(j4\phi_{1})$$

The solution of Equation (10) is $\theta_{\omega }=\phi_{1}+\frac{k\pi }{4},\;k=1,2\ldots$. The solution means that the effect on the axial declination angle of the main stiffness axis and frequency splitting is the same when adding mass at any position with a circumferential interval of 90°. In addition, because $\Delta =-\lambda_{m}m_{1}$, and $\lambda_{m}$ is calibrated to $\lambda_{m}=1.28\;\mathrm{Hz}/\mu g$ during the experiment, the amount of additional mass required for the adjustment can be calculated.

In the case of mass removal trimming, the original $-m_{1}$ in Equation (10) needs to be replaced with $m_{1}$.

(11) $$\Delta \exp(j4\theta_{\omega })=\lambda_{m}m_{1}\exp(j4\phi_{1})=-\lambda_{m}m_{1}\exp(j4\phi_{1}+\pi )$$

The above Equation (11) implies that the trimming effect is equivalent when the same mass is removed from the low-frequency axis at a 45° distance from the high-frequency axis.

## 3. Research on the Method of Adjusting Mass

### 3.1. The Method of Mass Adding Based on Gold Spheres Bonding

Within the micro-nano-scale, there are several methods of adding mass, such as focused electron beam-induced deposition (FEBID) [17], the laser-induced forward transfer (LIFT) [18] and common sputtering coating. However, the disadvantages of these methods are obvious, including but not limited to the complicacy of operation, expensiveness of equipment, and the limitation of added mass. Therefore, this study employs the bonding method to add gold spheres to the mass blocks that are coated with gold film. This method can easily and quickly complete the addition of mass, and the cost is low.

The principle of adding gold spheres is as follows. The first step is the formation of a sphere. When the gold wire leads out of the top part of the porcelain nozzle, the electronic ignition system generates a high voltage of several thousand volts. This voltage ionizes the air surrounding the electrode and the lead. The air between the electrode and the end face of the lead is broken down, forming an arc. The high temperature produced melts the tail wire of the gold wire. At the same time, under the action of gravity and surface tension, a seed sphere will be formed at the end of the wire. Then, there is the bonding of the gold sphere. The method used in the experiment is ultrasonic bonding, which involves the mechanical vibration of ultrasonic frequency between the bonding head and the gold film while applying pressure. The vibration can destroy the oxide layer on the surface of the gold film and generate heat. Under the combined action of heat, ultrasonic energy, and bonding force, atomic migration occurs between the gold sphere and the gold film, resulting in a strong connection at the bonding points.

**Figure 4:**
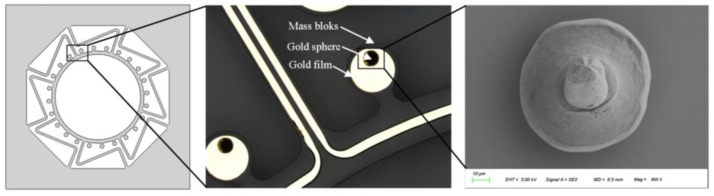
The addition of gold spheres.

The F&S semi-automatic wire bonding machine is engaged. The bonding pressure is 50–60 cN, the ultrasonic time is 35 ms, and the energy is 60–70 dig. The position and bonding effects are shown in Figure 4. The gold sphere is in the shape of a hemisphere with a diameter of about 70–80 μm.

### 3.2. The Method of Mass Removal Based on Femtosecond Laser

The two-temperature model is the theoretical basis of metal damage under the action of femtosecond laser ultra-short pulses [19]. That is, the energy transfer process during the interaction between the laser pulse and metal is divided into two steps: an electron and lattice-heating two-temperature process [20]. The damage mechanism of gold film based on this model has been investigated [21]. In this study, the ablation threshold of gold by a femtosecond laser is investigated experimentally based on the aforementioned theory. The effects of single-pulse energy, repetition frequency and processing speed on the ablation threshold are analyzed. In addition, the ablation threshold of fused silica and the morphology of gold spheres after ablation are also observed, which provide laser process parameters for mass removal trimming.

**Figure 5:**
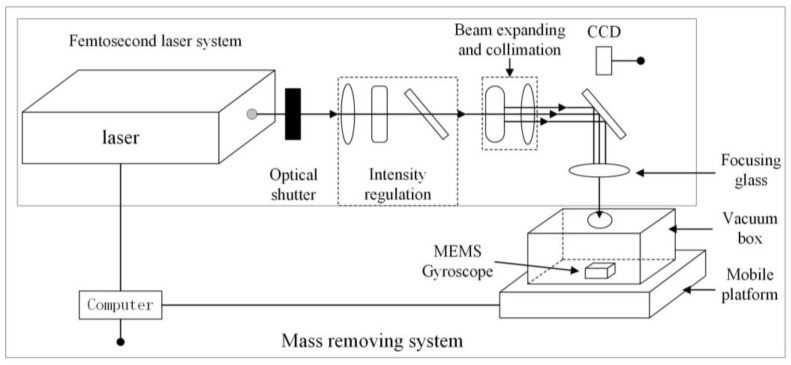
The femtosecond laser trimming system of mass removing.

The PHAROS femtosecond laser system from LIGHT CONVERSION, Lithuania, which possesses a wavelength of 343 nm, a Gaussian distribution of the spot, and a pulse width of 220 fs is employed. The diagram of the system is shown in Figure 5. The substrate of the experimental sample is fused silica, and the surface is sequentially plated with 10 nm of Cr and 300 nm of Au using magnetron sputtering.

#### 3.2.1. Influence of the Single-Pulse Energy

By controlling the laser repetition frequency of 200 kHz, the scanning speed of the vibrating mirror at 120 mm/s and other parameters, the single-pulse energy was adjusted by changing the power percentage from 3.2 to 20 watts in increments of 0.4 watts. Correspondingly, the single-pulse energy was changed from 15.8 to 98.6 μJ. The surfaces of the samples were etched and processed in a straight line one by one, and the results were observed by SEM.

**Figure 6:**
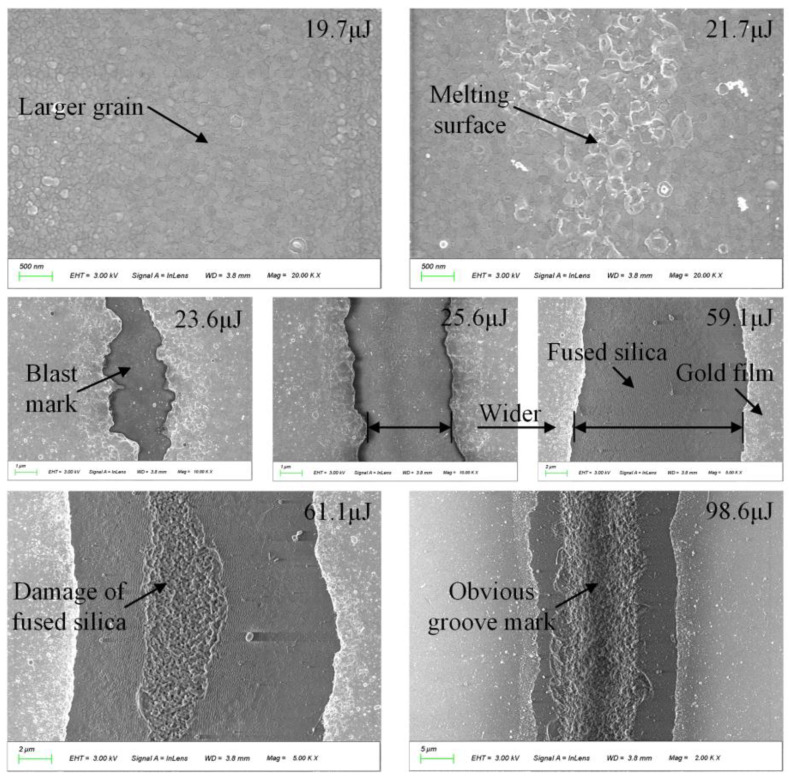
The change process of sample surface with single-pulse energy.

Figure 6 illustrates the change process of the sample surface with single-pulse energy. When the single-pulse energy of the laser is small, the dislocation defects are eliminated via the energy exchange between electron colliding with the lattice, resulting in the formation of large grains in the gold film within the irradiated area. With the energy increasing, the surface becomes molten. When exceeding the ablation threshold, part of the gold film is gasified, and the fused silica substrate is exposed. With the energy further increasing, the ablation area becomes larger, and the linewidth increases to about 15 μm at 59.1 μJ energy. Furthermore, when the single-pulse energy reaches 61.1 μJ, the fused silica substrate begins to be damaged. Finally, when the laser power reaches 100%, the energy of 98.6 μJ causes noticeable groove damage to the surface of fused silica.

Based on the above-mentioned results, the single-pulse energy should be controlled between 23.6 and 59.1 μJ to achieve the removal of the gold film and not damage the fused silica substrate. Subsequently, the energy parameter of 33.5 μJ is employed in the trimming process to remove the gold film.

#### 3.2.2. Influence of the Repetition Frequency

**Figure 7:**
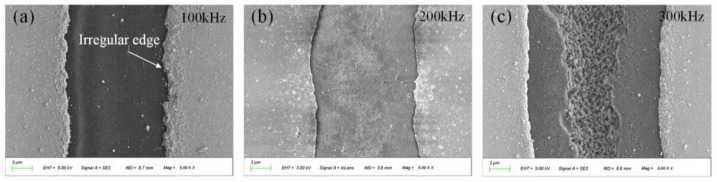
The ablative morphology at different repetition frequencies. (**a**) The ablative morphology at 100 kHz; (**b**) the ablative morphology at 200 kHz; (**c**) the ablative morphology at 300 kHz.

Compared to conventional single-pulse laser operation, a high-pulse repetition frequency can induce heat accumulation, which is more favorable for the ablation process. Figure 7 shows the surface ablation when the laser single-pulse energy is kept at 40 μJ and the repetition frequency varies between 100, 200, and 300 kHz. There are considerable differences among the results (gold film is removed at 100 kHz; the grain on the surface of the fused silica substrate starts to change at 200 kHz; the fused silica is ablated at 300 kHz).

#### 3.2.3. Influence of Processing Speed

**Figure 8:**
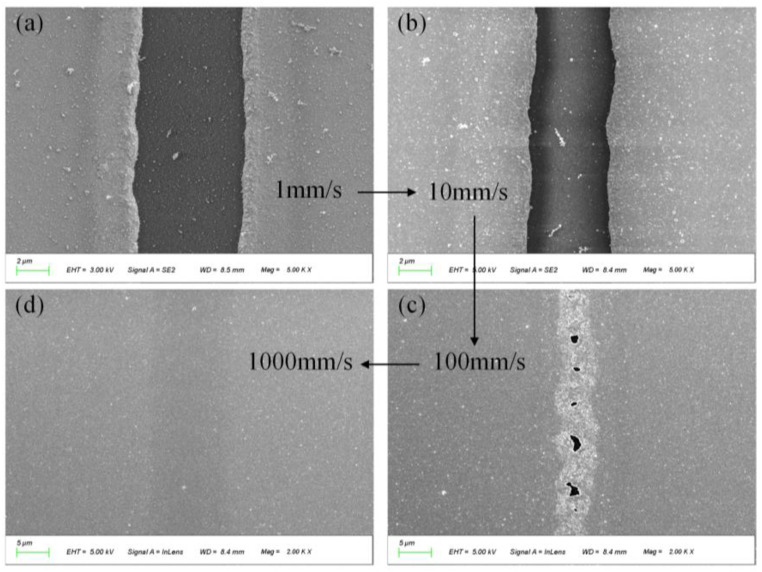
The ablative morphology at different processing speeds. (**a**) The ablative morphology at 1 mm/s; (**b**) the ablative morphology at 10 mm/s; (**c**) the ablative morphology at 100 mm/s; (**d**) the ablative morphology at 1000 mm/s.

The processing speed refers to the scanning speed of the oscillator during the processing of the image element, which can be described as the number of pulses per unit length. It is deemed that the processing speed influences the ablation threshold. Figure 8 shows the results of ablation at different speeds with an energy of 23.6 μJ. In brief, the slower the speed is, the lower the ablation threshold.

#### 3.2.4. Ablation Parameters of the Gold Sphere

In the previous three sections, the parameters of the laser used for removing gold film were analyzed, from which the parameters for removing gold spheres can be determined as well. First of all, the single-pulse energy is an important factor for ablation. Distinguished from the process of gold film, we have selected a slightly higher energy parameter of 39.4 μJ to remove more mass from the gold sphere while avoiding damage to the fused silica substrate. In terms of repetition frequency of the laser, increasing it while keeping the single-pulse energy constant leads to higher laser power, which in turn generates more heat. This increase in heat affects the accuracy of frequency split identification by amplifying the resonance frequency. When the repetition frequency is low, the surface regularity slightly deteriorates. To achieve better processing results, the repetition frequency of 200 kHz is employed. In terms of processing speed, a slower speed results in a higher number of pulses per unit length, generating more heat and impacting frequency. Additionally, the accumulation of heat and changes in the optical properties of the material affect the efficiency of removal, necessitating consideration. Therefore, we choose a processing speed of 120 mm/s.

**Figure 9:**
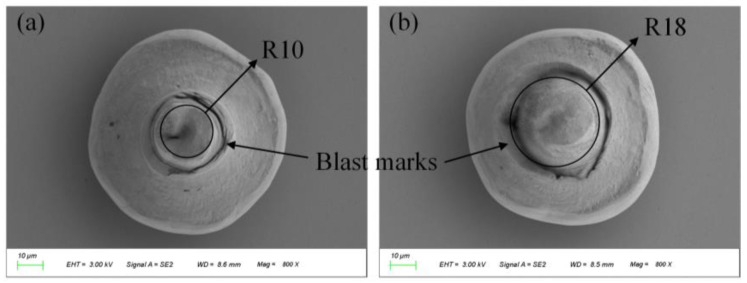
The morphology of the gold sphere after ablation. (**a**) The processing pattern is a disk with a radius of 10 μm; (**b**) the processing pattern is a disk with a radius of 18 μm.

After establishing the parameters of femtosecond laser ablation of the gold sphere, we opted for two processing patterns (discs with radii of 10 μm and 18 μm) to confirm removal of the gold sphere section, which we viewed via SEM, as shown in Figure 9. Compared to the image shown in Figure 4, it is evident that the gold sphere has been removed. As a result, this parameter will be utilized in the trimming process for the removal of the gold sphere.

## 4. Trimming Experiments

**Figure 10:**
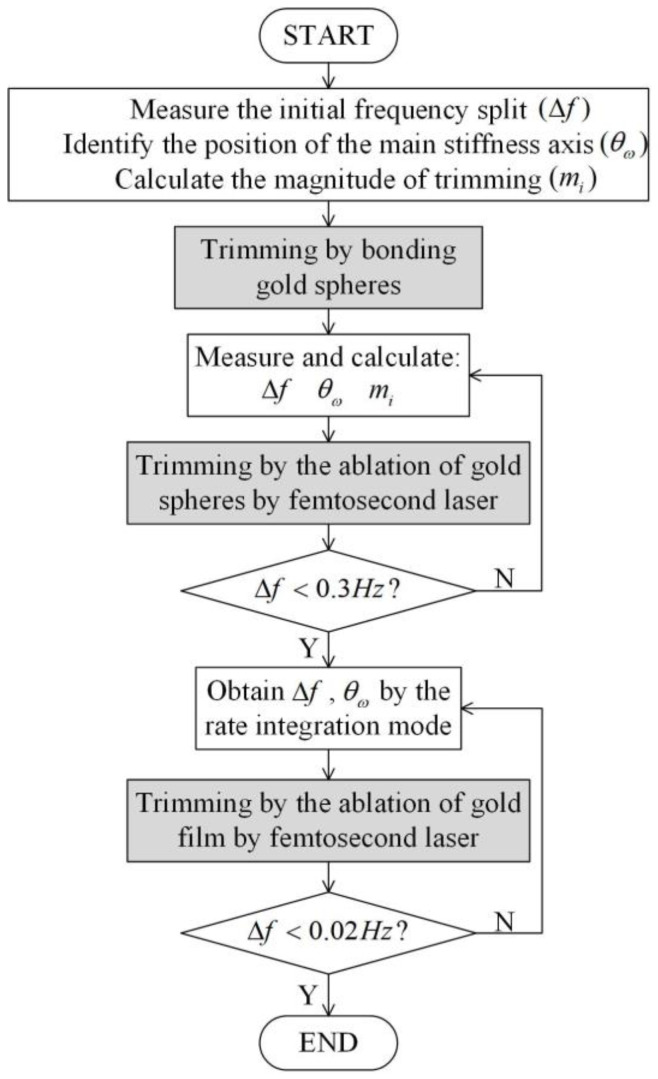
The flow chart of the three main stages of trimming.

The trimming method varies when the frequency splitting of the gyroscope is different. The trimming process includes three steps—adding the gold sphere, removing the gold sphere, and removing the gold film. The flowchart for trimming is shown in Figure 10. The method of adding gold spheres is suitable for initial trimming, as it provides a solution to the problem of insufficient mass removal during trimming when there is a large frequency split. The process of removing the gold sphere is termed rough trimming. The target is to decrease the frequency splitting to about 0.3 Hz. The last is the refinement process, in which the rate integral control method is applied to further reduce the frequency splitting by removing the gold film. The complete procedure takes two hours, inclusive of testing.

**Figure 11:**
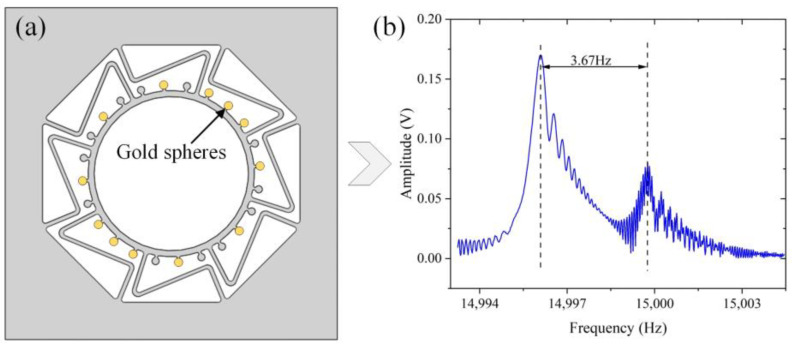
(**a**) The positions of added gold spheres; (**b**) the frequency splitting after adding gold spheres.

Before adding the gold sphere, the gyroscope’s frequency splitting of 4.36 Hz is measured by the amplitude–frequency curve. According to Equation (5) and Figure 3b, it is known that $\theta_{\omega }=\frac{1}{2}\arctan(\frac{X_{2}}{Y_{2}})\approx 0$, which implies that the high-frequency axis basically aligns with the position of the driving axis. Therefore, as shown in Figure 11a, gold spheres are added to each position at 8 positions among total 12 positions of the driving axis according to the central symmetry. Meanwhile, gold spheres are added to each of the four equivalent positions in the middle of the sensing axis for subsequent trimming after the deflection of the main stiffness axis.

**Figure 12:**
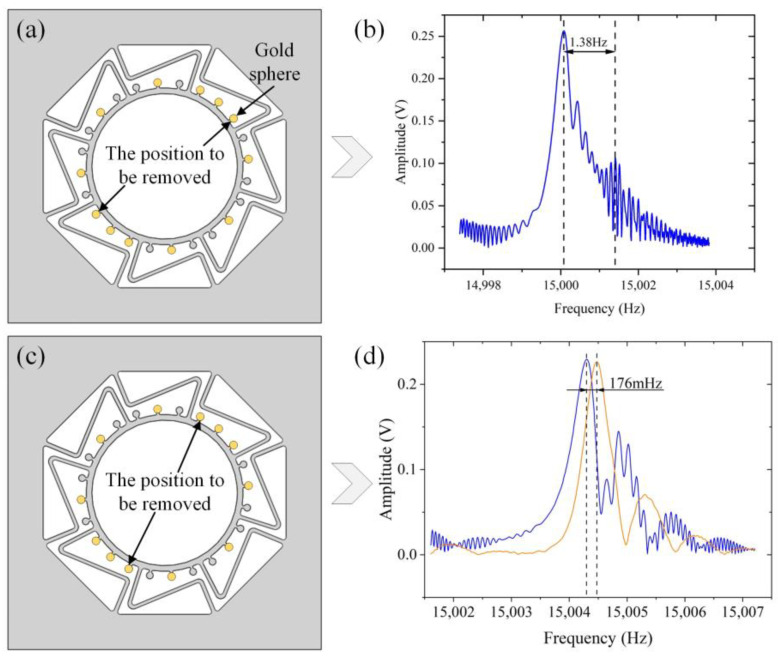
The process of rough trimming. (**a**) The positions of gold spheres to be removed. (**b**) The frequency split after removing a little mass. (**c**) The next positions of gold spheres to be removed. (**d**) The frequency split after the rough trimming.

Based on the added gold spheres, the frequency splitting is decreased to 3.67 Hz (Figure 11b). The driving axis changed from a high-frequency axis to a low-frequency axis, which indicates that not all of the added gold spheres were appropriate. Therefore, the next rough trimming process of removing gold spheres was conducted. According to Equation (5) and the amplitude–frequency curve shown in Figure 11b, the principal stiffness has been deflected. Because of the limitation on the number of raised mass blocks, the gold spheres closest to the low-frequency axis were ablated by a laser, as shown in Figure 12a. The processing pattern is a disk with a radius of 30 μm. The laser focus is adjusted 15 times, with a 5 μm drop each time, to ensure the desired amount of material removal. The trimming process reduces the frequency splitting to 1.38 Hz, as shown in Figure 12b.

After the aforementioned process, the low-frequency axis is deflected to the position shown in Figure 12c. Considering the small frequency splitting, the processing pattern varied into a disk with a radius of 18 μm while continuing the operation of laser removal of gold spheres. Finally, the frequency splitting is reduced to 176 mHz, as shown in Figure 12d.

**Figure 13:**
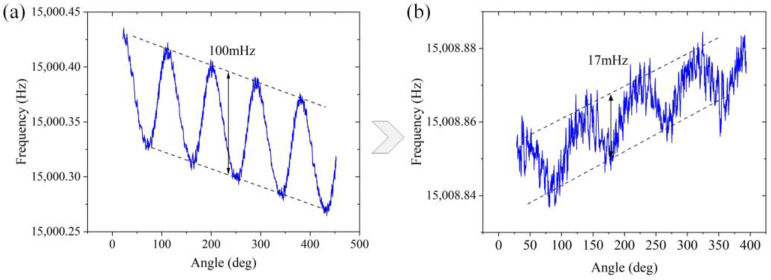
(**a**) The frequency split before refinement trimming; (**b**) the final frequency split of this gyroscope.

In this case, it is difficult to identify the position of the frequency splitting and the principal stiffness axis through the amplitude–frequency curve due to the frequency drift and other factors. Therefore, in the refinement stage, the gyroscope works in the rate integration mode. This working mode can only be realized when the frequency splitting is not large. The magnitude of frequency splitting and the position of the low-frequency axis are obtained via collecting the circumferential vibration frequency. The vibration frequency changes with the self-precession angle in a sinusoidal waveform, and the period is 90° when n=2 mode. The frequency splitting is the difference between the maximum and minimum values of the waveform. The angle corresponding to the trough represents the position of the low-frequency axis. That is, the place needs to be trimmed further. Then, the mHz-level trimming is implemented by removing the gold film by laser. The main parameters of the laser include a single-pulse energy of 33.5 μJ with the same repetition frequency (200 kHz) and a processing speed (120 mm/s). The result of the gyroscope after rough trimming is shown in Figure 13a. The actual frequency splitting is approximately 100 mHz. The result after trimming shows that the frequency splitting is minimized to 17 mHz, as shown in Figure 13b. In the medium vacuum environment, the quality factor is maintained at approximately 300,000 before and after the trimming process.

Based on the whole process, more experimental results are shown in Table 1. The frequency split of the gyroscope was reduced to about 20 mHz, respectively.

## 5. Conclusions and Prospect

This study proposes a combined method of adding mass and removing mass based on the error model and trimming model and applying it to trim the gyroscope, whose frequency splitting is 4.36 Hz. Following the steps of adding gold spheres at the high-frequency axis position and removing the gold at the low-frequency axis position, the frequency splitting is improved to 0.017 Hz. This achievement lays a foundation to improve the performance of the gyroscope. During the process, the ablation threshold of gold film by a femtosecond laser was also investigated. The corresponding main laser parameters for gold removal without damaging the fused silica substrate were determined.

However, the precision and efficiency of the proposed trimming method are more likely to be further improved if the amount of gold spheres added and removed can be controlled precisely. In addition, the theory of non-equivalent position combination trimming should also be investigated and applied as the basis to solve the limitation of the number of trimming positions, which may further improve the accuracy of trimming.

## Figures and Tables

**Table 1:** More experimental data on trimming.

Number	Initial Frequency Split	Final Frequency Split
Gyroscope 1	3.92 Hz	19 mHz
Gyroscope 2	3.58 Hz	21 mHz
Gyroscope 3	6.91 Hz	21 mHz

## Data Availability

The data that support the findings of this study are available from the corresponding author, Q.L., upon reasonable request.
